# Registration of histopathology to magnetic resonance imaging of prostate cancer

**DOI:** 10.1016/j.phro.2021.03.004

**Published:** 2021-04-12

**Authors:** Kristina Sandgren, Erik Nilsson, Angsana Keeratijarut Lindberg, Sara Strandberg, Lennart Blomqvist, Anders Bergh, Bengt Friedrich, Jan Axelsson, Margareta Ögren, Mattias Ögren, Anders Widmark, Camilla Thellenberg Karlsson, Karin Söderkvist, Katrine Riklund, Joakim Jonsson, Tufve Nyholm

**Affiliations:** a Department of Radiation Sciences, Radiophysics, Umea University, Sweden; b Department of Radiation Sciences, Diagnostic Radiology, Umea University, Sweden; c Department of Molecular Medicine and Surgery, Karolinska Institute, Stockholm, Sweden; d Department of Medical Biosciences, Pathology, Umea University, Sweden; e Department of Surgical and Perioperative Sciences, Urology and Andrology, Umea University, Sweden; f Department of Radiation Sciences, Oncology, Umea University, Sweden

**Keywords:** PET/MRI, Prostate cancer, Image registration, Histopathology correlation

## Abstract

**Background and purpose:**

The diagnostic accuracy of new imaging techniques requires validation, preferably by histopathological verification. The aim of this study was to develop and present a registration procedure between histopathology and *in-vivo* magnetic resonance imaging (MRI) of the prostate, to estimate its uncertainty and to evaluate the benefit of adding a contour-correcting registration.

**Materials and methods:**

For twenty-five prostate cancer patients, planned for radical prostatectomy, a 3D-printed prostate mold based on *in-vivo* MRI was created and an *ex-vivo* MRI of the specimen, placed inside the mold, was performed. Each histopathology slice was registered to its corresponding *ex-vivo* MRI slice using a 2D-affine registration. The *ex-vivo* MRI was rigidly registered to the *in-vivo* MRI and the resulting transform was applied to the histopathology stack. A 2D deformable registration was used to correct for specimen distortion concerning the specimen’s fit inside the mold. We estimated the spatial uncertainty by comparing positions of landmarks in the *in-vivo* MRI and the corresponding registered histopathology stack.

**Results:**

Eighty-four landmarks were identified, located in the urethra (62%), prostatic cysts (33%), and the ejaculatory ducts (5%). The median number of landmarks was 3 per patient. We showed a median in-plane error of 1.8 mm before and 1.7 mm after the contour-correcting deformable registration. In patients with extraprostatic margins, the median in-plane error improved from 2.1 mm to 1.8 mm after the contour-correcting deformable registration.

**Conclusions:**

Our registration procedure accurately registers histopathology to *in-vivo* MRI, with low uncertainty. The contour-correcting registration was beneficial in patients with extraprostatic surgical margins.

## 1 Introduction

In prostate cancer (PCa), imaging techniques such as magnetic resonance imaging (MRI) and positron emission tomography (PET) are used for advanced lesion characterization [1] and imaging-guided tailored treatment, whether it is surgical or the currently experimental method of boosting the dominant lesion during radiotherapy [2]. To incorporate advanced imaging techniques into the clinical management, validation of the diagnostic accuracy is required. For localized PCa, imaging findings may be validated by tissue samples from core needle biopsies. Tissue samples can be collected according to a standard biopsy template or targeted directly to the suspected lesion. A drawback of the systematic biopsies approach is the risk of missing relevant lesions, while with targeted biopsies only the suspected lesion can be validated, and consequently, no information about the remaining prostate is provided [3]. In imaging studies, a less biased validation method is to compare imaging findings with histopathology of a surgically removed specimen. This is preferably done by direct comparison with whole-mount histopathology [4] using image registration [5].

Image registration is a broad concept which in its simplest form involves spatially transforming one image to another, i.e., placing the images in the same frame of reference. The methods can vary from simple manual alignment based on a visual assessment to rigid or even deformable registrations based on computer-based optimizations of a variety of metrics [6], [7], [8], [9], [10], [11], [12], [13], [14], [15], [16]. When co-registering histopathology to *in-vivo* images, differences in image feature visibility due to resolution and contrast differences between the images may complicate the registrations. To compensate for this, an intermediate between the two different main image types may be used, e.g., an *ex-vivo* image of the surgical specimen. This has been described in several publications [6], [7], [11], [17], [18]. The *ex-vivo* image shows the same object, the specimen, as the histopathology image with image contrast similar to the *in-vivo* image but with increased resolution, given that they were obtained using the same imaging modality. The image registration process is further complicated by the fact that the images are not necessarily collected in the same imaging plane [5]. To address this, gross sectioning should be performed in the same orientation as the *in-vivo* image acquisition. One common technique to solve this problem is to use a device that fixates the specimen in the desired orientation and enables evenly distributed slices in the preferred plane [10], [11], [12], [13]. Further developments of this method use 3D-printer technology to create individually designed sectioning boxes, or prostate molds [19]. Shah et al. [20] published a method including a mold constructed by removing the *in-vivo* shape of the prostate from a pre-made box-shaped model, designed with thin slits evenly spaced for histopathology slicing. Similar designs have been used by a number of authors [9], [14], [21], [22]. Additionally, a prostate mold will likely improve the preservation of the *in-vivo* shape of the prostate that otherwise easily gets deformed after surgery. Scanning the specimen inside the mold will therefore improve the quality of the intermediate *ex-vivo* image, making it representative of the *in-vivo* image now both in shape and contrast. Resultingly, the *ex-vivo* image can be used to correct histopathology for specimen distortions occurring during and after histopathology sectioning.

This study aimed to develop and present a registration procedure between whole-mount histopathology and *in-vivo* PET/MRI of the prostate and to characterize its uncertainty. We also aimed to evaluate the benefit of using a contour-correcting deformable registration of the histopathology in the registration procedure.

## 2 Materials and methods

### 2.1 Patients

Twenty-five consecutive patients with intermediate and high-risk PCa, planned for laparoscopic radical prostatectomy at Umeå University Hospital, were included. Ethical approval was granted (Dnr 2016-229-31M) by the Regional Ethics Board and the Radiation Protection Committee at Umeå University Hospital. All patients signed informed consent to participate in the study. Inclusion criteria were histologically confirmed intermediate or high-risk PCa planned to be treated with radical prostatectomy, ≥2 months since the last prostate biopsy, Gleason score 3 + 4 or higher, written informed consent, and age >18 years. Exclusion criteria were contraindication to PET or MRI (non-MRI-safe implants, claustrophobia, physical limitations e.g., back pain), neoadjuvant/concomitant androgen deprivation therapy (medical or surgical castration; anti-androgens), TUR-P performed within 6 months, metastatic disease, and creatinine clearance <30 ml/min.

### 2.2 *In-vivo* imaging

All patients were examined with PET/MRI (SIGNA PET/MR 3T, GE Healthcare, Milwaukee, WI USA) before radical prostatectomy. The mean number of days between imaging and surgery was 35 (range: 5–132 days). The MRI-protocol was a diagnostic multiparametric pelvic protocol, including morphological three-plane T2-weighted (T2W) sequences (transaxial, coronal, and sagittal). MRI-parameters are shown in Supplementary Table 1A.

**Fig. 1:**
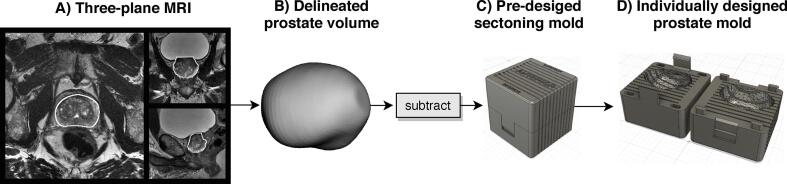
Creation of prostate molds; Flowchart of how an individually designed prostate mold, used in this study, is created. A) A three-plane T2W MRI is used to delineate the volume of the prostate (white ROI). B) The delineated prostatic volume is subtracted from a pre-designed mold (C) that consists of two parts constructed with a locking mechanism holding the box together (D).

Based on the three-plane T2W MRI, the prostatic volume was delineated in RayStation version 4.5 (RaySearch Laboratories AB, Stockholm, Sweden) by a medical physicist (either KS or JJ) and reviewed by a radiologist (SS). This delineation was used as input when finalizing the structure using the model-based segmentation tool in RayStation (Fig. 1A). From this, two structures were created by adding a margin of 1 and 2 mm, respectively, and exported as DICOM radiotherapy (RT)-structs.

### 2.3 3D-printed individually designed prostate molds

Individually designed prostate molds were created for each patient to guide histopathology sectioning and to preserve the *in-vivo* shape and orientation of the specimen. A cubical shape was created in Fusion 360 (Autodesk, Inc. San Rafael, California, USA) (Fig. 1C). The cube had a standard volume of 6.6 × 6.6 × 6.6 cm^3^ and was constructed out of two separate parts that could be joined using a locking mechanism (Fig. 1D). Throughout the cube, eleven 1 mm thick slits were inserted (with 5 mm spacing), to be used for histopathology slicing. The exported RT-structs were converted into Standard Triangle Language (STL)-files using MICE toolkit (Nonpi Medical AB, Umeå, Sweden) [23]. The STL-files were imported into Meshmixer (Autodesk, Inc. San Rafael, California, USA) where they were smoothed and simplified by reducing the number of vertices (Fig. 1B) and subsequently used as inputs into Fusion 360 and subtracted from the cube. For each patient two molds were printed, with a margin of +1 mm and +2 mm, respectively, using MakerBot Replicator + 3D-printer (MakerBot Industries, Brooklyn, NY USA).

### 2.4 *Ex-vivo* MRI of the specimen

**Fig. 2:**
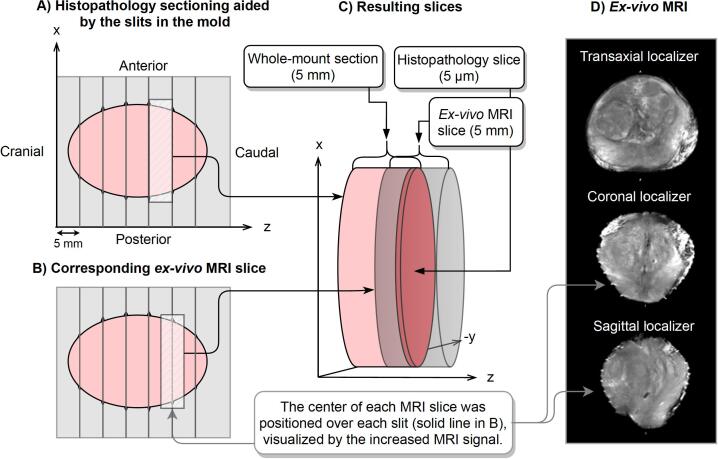
Slice location – histopathology and ex-vivo MRI; A) Illustrates specimen sectioning using the slits in the prostate mold. B) The corresponding ex-vivo MRI slices, centered over the guiding slits in the mold. C) The resulting overlap between whole-mount section (pink), ex-vivo MRI slice (gray), and histopathology slice (darker pink). D) The ex-vivo MRI localizers, where the coronal and sagittal were used when positioning the ex-vivo MRI slices, guided by increased MRI signal in the slits in the mold (illustrated as spikes in the ellipsoids). (For interpretation of the references to color in this figure legend, the reader is referred to the web version of this article.)

Immediately following surgery, an experienced research nurse removed the seminal vesicles and color-labeled the specimen for laterality before it was placed in the +1 mm margin mold. The posterior part of the prostate was colored in yellow and the right and left sides were colored in green and red, respectively. If the mold could not be closed without compressing tissue, the mold with a +2 mm margin was used. Using the same PET/MRI scanner, high-resolution *ex-vivo* T2W imaging of the specimen was performed before formalin-fixation (<90 min post-resection). Transaxial, sagittal, and coronal localizers were acquired to aid in the positioning of the MRI slices (Fig. 2). All *ex-vivo* MRI slices were positioned with their center located over the outermost caudal side of the histology section (Fig. 2B). Increased MRI-signal originating from fluid in the slits, visible in the localizers, was used to guide the slice positioning. MRI-parameters are shown in Supplementary Table 1A.

### 2.5 Histopathology preparations

The specimen was fixed in formalin at least 24 h before sectioning. Sectioning was subsequently performed by a pathologist using the slits in the mold, into 5 mm thick sections from the apex to the base. The histopathology examination included the standard procedure of pathologic-anatomic diagnosis analysis (dehydration, paraffin embedding, and microtome sectioning in 5 µm thick slices starting from the caudal surface of each paraffin block). The coloration of the prostate enabled the slices to be oriented correctly anatomically by examining the color of their edges. Each slice was glass-mounted and stained with hematoxylin and eosin. The first apical slice was, according to clinical routine, divided into several small pieces to enable examination of the apical prostate surface and could therefore not be used in this analysis.

### 2.6 Registration workflows

**Fig. 3:**
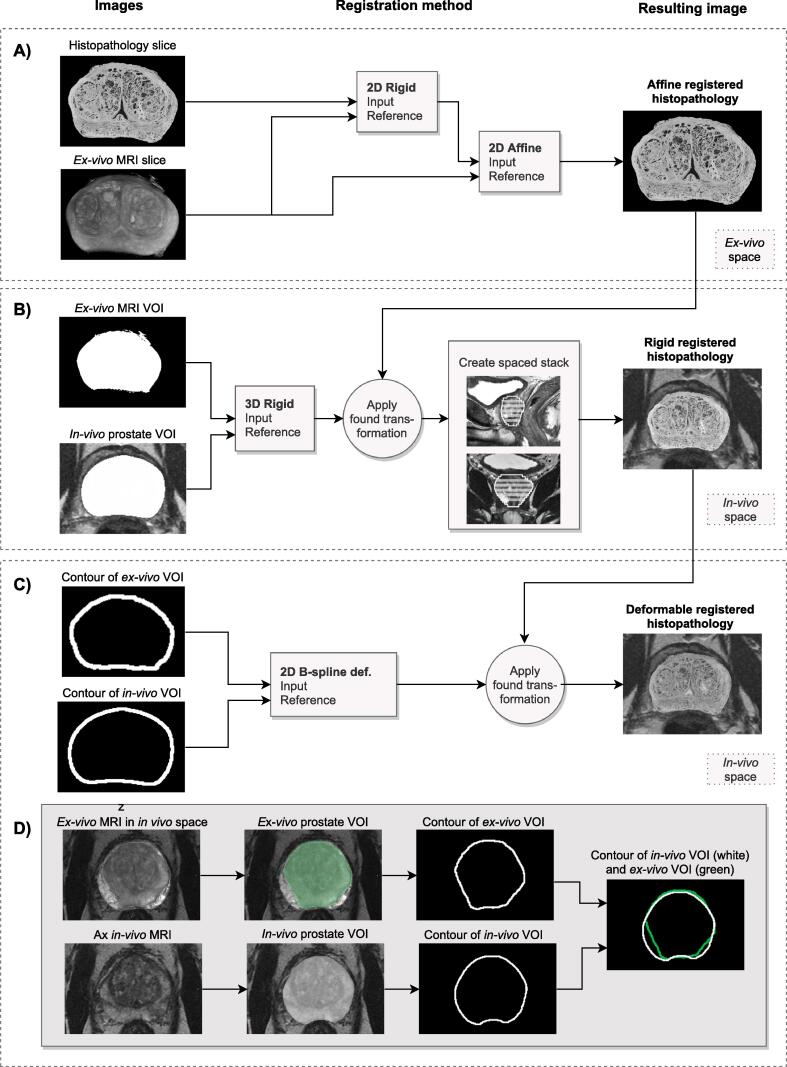
The registration procedure; A flowchart of the process to register histopathology to in-vivo PET/MRI data. A) The histopathology slice is rigidly registered to its corresponding ex-vivo MRI before they are registered with a 2D affine registration. B) A 3D rigid registration is applied to move the ex-vivo MRI to the in-vivo MRI, by using the pre-defined in-vivo prostatic volume and a mask of the ex-vivo MRI volume. C) The contours of the ex-vivo MRI were registered to the defined in-vivo MRI volume to correct for tissue distortions occurring when the specimen was in the prostate mold. D) Shows an example of a patient with large extraprostatic margins after surgery. The green ROI represents the ex-vivo contour and the white ROI represents the delineated prostatic volume from the transaxial in-vivo MRI. (For interpretation of the references to color in this figure legend, the reader is referred to the web version of this article.)

All registrations were done in MICE toolkit [23] which uses a software package for image registration, Elastix [24], based on the Insight Toolkit [25] code. The *in-vivo* transaxial (Ax) T2W MRI was used as the reference image, i.e., to which all other images were registered. A flowchart of the registration procedure can be seen in Fig. 3.

Each histopathology slice was first manually paired to its corresponding *ex-vivo* MRI slice through a visual assessment before a 2D-affine registration using mutual information as the similarity-measure was performed to account for specimen shrinkage and shearing that might have occurred during the pathology preparations. In some patients when the resection margin (often clearly seen on the *ex-vivo* MRI slice) was not visible in the histopathology image, or if the histopathology slice was not intact, only the intact parts were used for registration to the *ex-vivo* image. In patients with a prostate larger than the size of a standard glass-slide, the pathologist divided it into two parts. These parts were combined manually before registration (Fig. 3A). The *ex-vivo* MRI was registered to the *in-vivo* Ax T2W MRI using a 3D-rigid registration, and the registered histopathology stack was transformed accordingly. This registration was a mask-to-mask registration between the binary mask covering the prostatic volume of the *ex-vivo* MRI and the mask delineating the *in-vivo* prostatic volume. Mean squared difference was used as a similarity metric. We assumed that the prostate was fixed inside the mold, and therefore only rotations around the z-axis were allowed in the registration. The images differed in slice thickness and after registration, each histopathology and *ex-vivo* MRI (5 mm) covered two *in-vivo* MRI slices (2.5 mm). We reconstructed the registered stacks with 2.5 mm slice thickness and 2.5 mm spacing (Fig. 3B). Furthermore, a contour-correcting deformable registration was performed to account for specimen distortion that might occur before or during the period in which the specimen was inside the prostate mold. Possible reasons for these distortions could be extraprostatic resection margins, less margin than expected, or if the delineation of the prostate (used for mold creation) was incorrect leading to small specimen distortions of the specimen inside the mold. To correct these distortions, a 2D non-rigid B-spline registration registering the actual prostatic contour of the *ex-vivo* MRI volume (e.g., without extraprostatic margins) to the contour of the delineated *in-vivo* prostate. This registration was a multimetric registration using mean squared difference and bending energy penalty (weighted: 0.9999/0.0001). The control point spacing of the b-spline transformation was 10 mm. In all patients, a visual inspection of the delineated *in-vivo* prostate, used for mold creation, was done and if improvements were possible, the delineation was adjusted manually before the registration (Fig. 3C).

### 2.7 Uncertainty estimation

The uncertainty was estimated by measuring the distance between anatomical landmarks in the *in-vivo* Ax T2W MRI and the corresponding registered histopathology image. All landmarks were first identified in the *in-vivo* Ax T2W and included only if the corresponding structures was identified in the registered histopathology image. Landmarks were defined by drawing ROIs including the entire landmark, and their mass center positions were recorded. The absolute error was calculated in x- and y-directions and reported together with their combined in-plane error based on the Euclidean distance, $\sqrt{{x^{2}+y}^{2}}$. To study the effect of the contour-correcting deformable registration, all landmarks were delineated before and after this registration. The patients were divided into two groups, one including patients with visually apparent extraprostatic surgical margins, and the other one including patients without.

## 3 Results

Eighty-four landmarks were identified in the 25 patients when evaluating the uncertainty of the registration procedure. Landmarks were located in the urethra (62%), cysts at various positions in the prostate (33%), and the ejaculatory ducts (5%). The median number of identified landmarks was 3 per patient (range: 1–7), Supplementary Fig. 1A shows the number of landmarks, and their anatomical location, for all patients. Fig. 5 shows an example of the mass center of a landmark defined in the in-vivo MRI and corresponding registered histopathology slice.

**Fig. 4:**
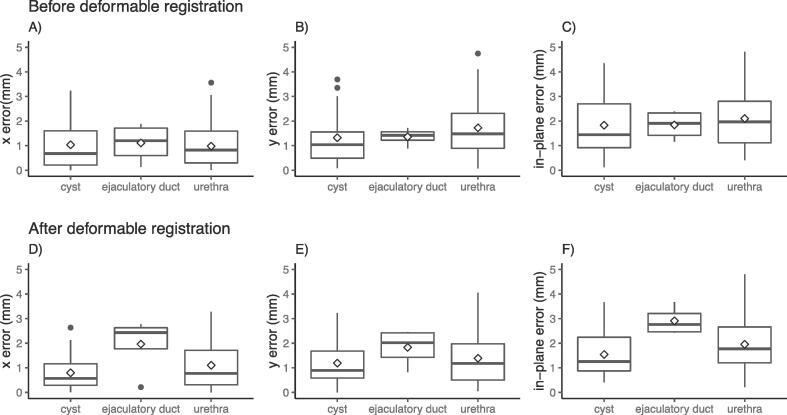
Resulting uncertainties; The resulting x-, y-, and in-plane error when comparing landmarks identified in the in-vivo transaxial MRI and the registered histopathology. (A–C) Shows the result before and (D–F) after contour-correcting deformable registration. Each box represents the interquartile range (IQR), i.e., the first and third quartile. The horizontal line in each box defines the median value and the diamond the mean value. The top whisker is calculated by adding 1.5 * IQR to the third quartile, and the bottom whiskers by subtracting 1.5 * IQR from the first quartile. Black points represent data points located outside the end of each whisker.

The median in-plane error [interquartile range (IQR)] was estimated to 1.8 [1.2, 2.7] mm before and 1.7 [1.0, 2.5] mm after the contour-correcting deformable registration. The median error in the x-direction was estimated to 0.8 [0.3, 1.6] mm before and after the contour-correcting deformable registration. In the y-direction, the median error was 1.3 [0.8, 2.7] mm before and 1.1 [0.6, 2.0] mm after contour-correcting deformable registration. Fig. 4 shows these results graphically, separated by the anatomical location of the landmark. In the ejaculatory ducts, located in the outmost part towards the prostatic base, the uncertainty increased after the contour-correcting deformable registration, while the other anatomical sites did not change markedly.

**Fig. 5:**
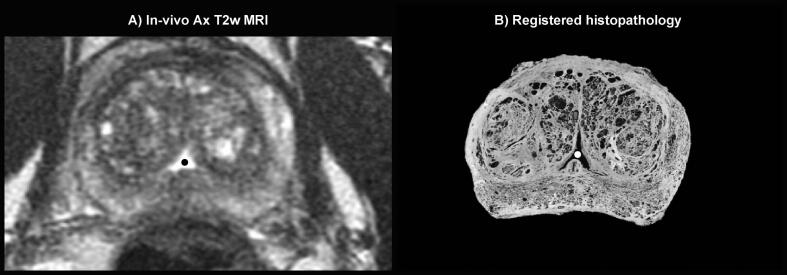
Example of a landmark; In-vivo MRI slice including the mass center (black dot) of the landmark defined as urethra in this slice (A), and the corresponding registered histopathology slice including corresponding landmark in urethrea (white dot) (B).

Comparing the results of group 1 (14 patients with extraprostatic surgical margins) and group 2 (11 patients without extraprostatic surgical margins), group 1 had a median in-plane error of 2.1 [1.4, 3.2] mm before and 1.8 [1.2, 2.6] mm after contour-correcting deformable registration. In group 2, the median error was 1.3 [0.9, 2.4] mm before and 1.5 [1.0, 2.5] mm after the contour-correcting deformable registration. Supplementary Table 2A shows the resulting data from groups 1 and 2.

## 4 Discussion

New imaging techniques require validation, preferably by histopathological verification, to ensure their diagnostic accuracy. We present a registration procedure that registers histopathology to *in-vivo* MRI data. Several authors have tackled this, however, to our knowledge, only Reynolds et al. [11] have published a methodology that includes both *ex-vivo* MRI of the specimen and guided histopathology sectioning. Our concept is based upon this method, but unlike Reynolds et al. who used a sectioning-box, we used individually designed prostate molds, as described by Shah et al. [20]. Supplementary Table 3A summarizes similar studies, their registration methods, evaluation methods, and uncertainties. An established repeatable evaluation method in studies of this kind is to measure distances between landmarks, and our results add to the support for this method when compared to previous publications (ranging from 1.1 mm [7] to 3.2 mm [11]).

In this study, we transform the histopathology by an affine transform to fit the *ex-vivo* MRI shape, and to correct specimen distortion (shrinkage and shearing) that may have occurred during the histopathology processing. This registration uses the boundary and internal structures of the *ex-vivo* MRI and histopathology image, two images with high resolution and detailed internal structures, to find the optimal transform. When registering the *ex-vivo* MRI volume to the *in-vivo* Ax T2W, we found that registering the volumes directly to each other resulted in a less robust method compared to using a mask-to-mask registration. This is likely due to the fact that the *in-vivo* MRI has lower resolution, and internal structures are sometimes hard to identify, compared to the *ex-vivo* MRI. For the same reason, we registered contours in our contour-correcting deformable registration. Since none of the steps in our registration procedure focus on landmarks in the *in-vivo* MRI we believe that comparing landmarks is a valid method for evaluating uncertainty. One observer (KS) identified all landmarks, which could have affected the result, but since only landmarks which clearly corresponded to each other were included, the risk of bias should be low.

We studied the uncertainty in the x-, y-direction, and the combined in-plane uncertainty. No apparent difference between the directions could be found. There was also no apparent difference between landmarks identified in the central parts of the prostate (urethra) compared to landmarks identified in varying locations within the prostate (cysts). However, we found an increased uncertainty in the ejaculatory ducts landmarks. A plausible explanation could be that the prostatic boundary is hard to define in the vicinity of the seminal vesicles, close to the ejaculatory ducts, and also that the tissue may be distorted upon removal of the seminal vesicles.

To ensure that the prostate was fixated inside the mold and to account for prostatic volume changes between imaging and surgery, two molds were printed for each patient. In all patients, the +1 mm mold was the first choice, and only if the mold could not be closed without compressing the tissue, the +2 mm mold was used. Each prostate was carefully positioned as accurately as possible in its mold to avoid any rotations, and we could therefore exclude rotations around the x- and y-axis in our 3D-rigid registration. With higher *in-vivo* T2W MRI resolution a possible methodological improvement would be to define the superior and inferior parts of the urethra and use as guidance when placing the specimen in its mold, which would likely reduce the uncertainty in our method slightly. In this study, the pathologist performed a visual assessment of the sections which did not reveal varying slice thickness, indicating that the specimens were sufficiently fixated in the mold. One concern is the uncertainty resulting from differences in slice thickness between histopathology (5 μm) and *ex-vivo* MRI (5 mm). Each histopathology slice is sectioned from the base of each whole-mount sections (5 mm) and hence only representing the outermost part of each whole-mount section. The MRI slices represent signals detected from the entire slice volume. To minimize the uncertainty in the z-direction we positioned all *ex-vivo* MRI slices so that the center of each MRI slice corresponded to the location of the histopathology slice (Fig. 2). When the *ex-vivo* MRI volume is rigidly registered to the prostate volume defined in the *in-vivo* MRI (2.5 mm), each *ex-vivo* MRI slice covers two or three *in-vivo* MRI slices.

In conclusion, our procedure accurately registers histopathology to *in-vivo* MRI data with a median in-plane error of 1.7 mm. We showed a benefit of using a contour-correcting deformable registration to correct for specimen distortions in patients where surgery was performed with extraprostatic margins.

## Declaration of Competing Interest

The authors declare the following financial interests/personal relationships which may be considered as potential competing interests: Authors Joakim Jonsson and Tufve Nyholm are part owners in Nonpi Medical AB. All other co-authors have no potential competing interest to report.
