# Supplementary material for: Registration of histopathology to magnetic resonance imaging of prostate cancer
Source: Phys Imaging Radiat Oncol. 2021 Apr 12;18:19–25. doi: 10.1016/j.phro.2021.03.004 (PMC8254194; doi:10.1016/j.phro.2021.03.004)
Supplement: Supplementary data 1 [file mmc1.pdf]

SUPPLEMENTARY MATERIALS

Table 1A: Characteristics of the different images used in the image registration procedure.

	***In-vivo* Axial**	***In-vivo* Coronal**	***In-vivo* Sagittal**	***Ex-vivo* Axial**	**Histopathology (in MICE)**	**Registered histopathology**
**Repetition time (ms)**	9946	6133	5793	2500	-	-
**Echo time (ms)**	103	128	128	117	-	-
**Flip angle (°)**	125	111	111	111	-	-
**Number of averages (NEX)**	1	3	3	15	-	-
**Slice thickness (mm)**	2.5	3	3	5	5 x 10^{-3}	2.5
**In-plane pixel size (mm^2)**	0.410 x 0.410	0.469 x 0.469	0.469 x 0.469	0.195 x 0.195	0.015 x 0.015	0.1 x 0.1
**Matrix size**	512 x 512	512 x 512	512 x 512	512 x 512	3840 x 1960	2101 x 2101

Table 2A: In-plane error (mm) of group 1 (14 patients with extraprostatic surgical margins) and group 2 (11 patients without extraprostatic surgical margins).

	**Min**	**1st Qu.**	**Median**	**Mean**	**3dr Qu.**	**Max**	**SD**
**Group 1** (before deformable registration)	0. 2	1.4	2.1	2.3	3.2	4.4	1.8
**Group 1** (after deformable registration)	0.4	1.2	1.8	2.0	2.6	4.8	1.1
**Group 2** (before deformable registration)	0.1	0.9	1.3	1.7	2.4	4.8	1.2
**Group 2** (after deformable registration)	0.2	1.0	1.5	1.7	2.5	4.3	1.0

Table 3A: Summary of other publications performing co-registration of histopathology to *in-vivo* MRI and determine their method uncertainty based on comparing positions of landmarks.

**Publication**	**Registrations scheme**	**Evaluation method**	**Uncertainty**
**Ward et al (2012)** [1]	Landmarks-based (by extrinsic fiducial markers) comparing rigid and nonrigid thin-plate spline registration of histopathology to *in-vivo* MRI.	Euclidian distance between corresponding manually determined landmarks in the *in-vivo* T2w MRI and registered histopathology sections.	2.3 ± 1.7 mm (rigid)<br>1.1 ± 0.7 mm (nonrigid)
**Orczyk et al (2012)** [2]	Landmark-based (by extrinsic fiducial markers) spatial registration (rigid and affine) of histopathology to *in-vivo* MRI.	The mean root square distance across corresponding control points defined in the registered histopathology and *in-vivo* MRI.	2.89 mm (rigid)<br>1.59 mm (affine)
**Kalavagunta et al (2015)** [3]	Local affine transformation assisted by internal structures (IS) of histopathology to *in-vivo* MRI.	Root mean squared distance between manually detected landmarks (independent from the ones used in the registration) defined in the *in-vivo* MRI and the native histopathology before transformed.	1.54 ± 0.64 mm (with IS)<br>2.92 ± 1.76 mm (without IS)
**Reynolds et al (2015)** [4]	Initial alignment followed by a deformable registration of *in-vivo* MRI to *ex-vivo* MRI.<br><br>Histopathology to *ex-vivo* MRI by a rigid registration followed by an initial control point alignment and automatic registration using similarity transform.	Mean distance between point features.<br><br>Quantitative assessment by computing the mean distance between control points after registration.	3.2 ± 1.3 mm<br><br>0.57 ± 0.28 (0.06–1.99) mm
**Uribe et al (2015)** [5]	An affine and a nonrigid transform of the *in-vivo* MRI to histopathology. The nonrigid was used to correct for deformation due to the usage of endorectal coil during *in-vivo* MRI.	Median distance between corresponding landmarks in the *in-vivo* MRI and registered histopathology sections.	1.55 (0.3-3.1) mm
**Dinh et al (2017)** [6]	Deformable registration method based on selected landmarks.	Average distance between landmark in the *in-vivo* MRI and registered histopathology.	2.1 mm (dataset 1)<br>2.6 mm (dataset 2)
**Li et al (2017)** [7]	Multi-scale spectral embedding registration of histopathology to *in-vivo* MRI.	Root mean square distance between corresponding landmarks in registered histopathology and *in-vivo* MRI.	2.96 ± 0.76 mm

REFERENCES

1. Ward AD, Crukley C, McKenzie CA, Montreuil J, Gibson E, Romagnoli C, et al. Prostate: registration of digital histopathologic images to in vivo MR images acquired by using endorectal receive coil. Radiology. 2012;263:856–864. http://www.ncbi.nlm.nih.gov/pubmed/22474671.
2. Orczyk C, Mikheev A, Rosenkrantz A, Melamed J, Taneja SS, Rusinek H. Imaging of prostate cancer: a platform for 3D co-registration of in-vivo MRI ex-vivo MRI and pathology. Proc SPIE-the Int Soc Opt Eng. 2012;8316:1–18. http://proceedings.spiedigitallibrary.org/proceeding.aspx?doi=10.1117/12.911369.
3. Kalavagunta C, Zhou X, Schmechel SC, Metzger GJ. Registration of in vivo prostate MRI and pseudo-whole mount histology using Local Affine Transformations guided by Internal Structures (LATIS). J Magn Reson Imaging. 2015;41:1104–1114.
4. Reynolds HM, Williams S, Zhang A, Chakravorty R, Rawlinson D, Ong CS, et al. Development of a registration framework to validate MRI with histology for prostate focal therapy. Med Phys. 2015;42:7078–7089.
5. Uribe CF, Jones EC, Chang SD, Goldenberg SL, Reinsberg SA, Kozlowski P. In vivo 3T and ex vivo 7T diffusion

tensor imaging of prostate cancer: Correlation with histology. Magn Reson Imaging. 2015;33:577–583. http://www.ncbi.nlm.nih.gov/pubmed/25721995.

6. Dinh CV, Steenbergen P, Ghobadi G, Poel H Van Der, Stijn WTPJ. Multicenter validation of prostate tumor localization using multiparametric MRI and prior knowledge. Med Phys. 2017;44:949–961. http://ovidsp.ovid.com/ovidweb.cgi?T=JS&PAGE=reference&D=emexa&NEWS=N&AN=615061982.

7. Li L, Pahwa S, Penzias G, Rusu M, Gollamudi J, Viswanath S, et al. Co-Registration of ex vivo Surgical Histopathology and in vivo T2 weighted MRI of the Prostate via multi-scale spectral embedding representation. Sci Rep. 2017;7:1–12. http://dx.doi.org/10.1038/s41598-017-08969-w.

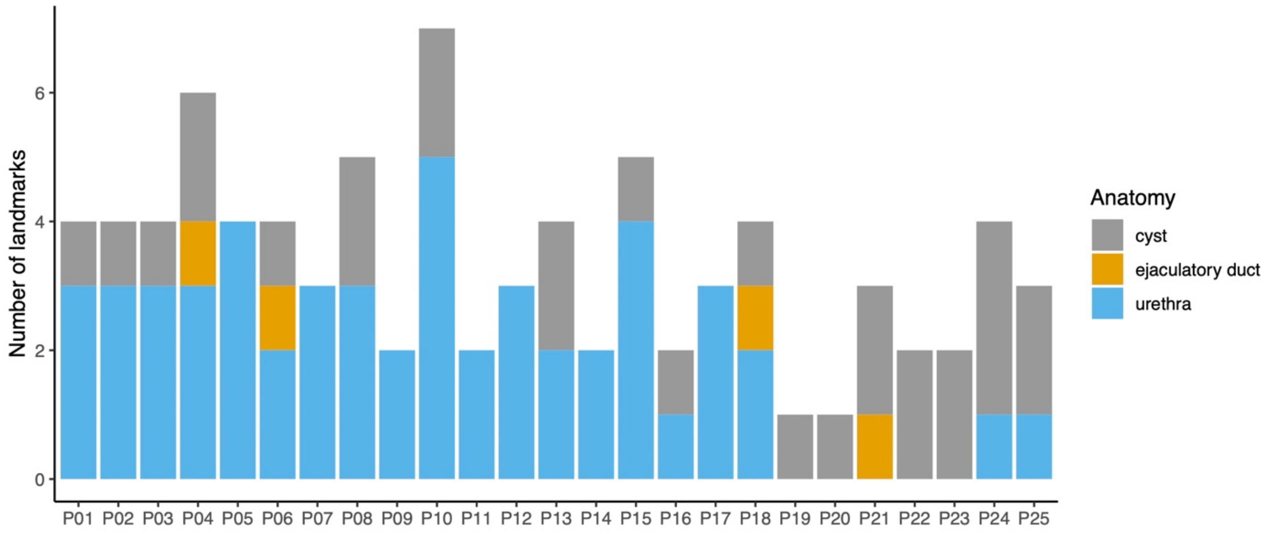

Figure 1A: Identified landmarks per patients and their anatomical location.
